# Disparities in Breast Cancer Diagnostics: How Radiologists Can Level the Inequalities

**DOI:** 10.3390/cancers16010130

**Published:** 2023-12-27

**Authors:** Filippo Pesapane, Priyan Tantrige, Anna Rotili, Luca Nicosia, Silvia Penco, Anna Carla Bozzini, Sara Raimondi, Giovanni Corso, Roberto Grasso, Gabriella Pravettoni, Sara Gandini, Enrico Cassano

**Affiliations:** 1 Breast Imaging Division, IEO European Institute of Oncology IRCCS, 20141 Milan, Italy; anna.rotili@ieo.it (A.R.); luca.nicosia@ieo.it (L.N.); silvia.penco@ieo.it (S.P.); anna.bozzini@ieo.it (A.C.B.); enrico.cassano@ieo.it (E.C.); 2 King’s College Hospital NHS Foundation Trust, London SE5 9RS, UK; p.tantrige@nhs.net; 3 Molecular and Pharmaco-Epidemiology Unit, Department of Experimental Oncology, IEO European Institute of Oncology IRCCS, 20141 Milan, Italy; sara.raimondi@ieo.it (S.R.); sara.gandini@ieo.it (S.G.); 4 Department of Oncology and Hemato-Oncology, University of Milan, 20122 Milan, Italy; giovanni.corso@ieo.it (G.C.); roberto.grasso@ieo.it (R.G.); gabriella.pravettoni@ieo.it (G.P.); 5 Division of Breast Surgery, IEO European Institute of Oncology IRCCS, Via Ripamonti, 435, 20141 Milan, Italy; 6 European Cancer Prevention Organization (ECP), 20122 Milan, Italy; 7 Applied Research Division for Cognitive and Psychological Science, IEO European Institute of Oncology IRCCS, 20141 Milan, Italy

**Keywords:** breast neoplasms, radiology, healthcare inequities, health policy, cultural competency, early detection of cancer, teleradiology, artificial intelligence

## Abstract

**Simple Summary:**

This paper delves into the persistent issue of unequal access to medical imaging, with a particular focus on breast cancer screening and its impact on marginalized communities and racial/ethnic minorities. Central to our discussion is the role of scientific mobility among radiologists in fostering healthcare policy changes that promote diversity and cultural competence. We propose various strategies to bridge this gap, including cultural education, sensitivity training, and diversifying the radiology workforce. These measures aim to improve communication with diverse patient groups and reduce healthcare disparities. Additionally, we explore the challenges and advantages of teleradiology as a means to extend medical imaging services to underserved areas. In the context of artificial intelligence, we emphasize the critical need to validate algorithms across diverse populations to ensure unbiased and equitable healthcare outcomes. Overall, this paper underscores the importance of international collaboration in addressing global access barriers, presenting it as a key to mitigating disparities in medical imaging access and contributing to the pursuit of equitable healthcare.

**Abstract:**

Access to medical imaging is pivotal in healthcare, playing a crucial role in the prevention, diagnosis, and management of diseases. However, disparities persist in this scenario, disproportionately affecting marginalized communities, racial and ethnic minorities, and individuals facing linguistic or cultural barriers. This paper critically assesses methods to mitigate these disparities, with a focus on breast cancer screening. We underscore scientific mobility as a vital tool for radiologists to advocate for healthcare policy changes: it not only enhances diversity and cultural competence within the radiology community but also fosters international cooperation and knowledge exchange among healthcare institutions. Efforts to ensure cultural competency among radiologists are discussed, including ongoing cultural education, sensitivity training, and workforce diversification. These initiatives are key to improving patient communication and reducing healthcare disparities. This paper also highlights the crucial role of policy changes and legislation in promoting equal access to essential screening services like mammography. We explore the challenges and potential of teleradiology in improving access to medical imaging in remote and underserved areas. In the era of artificial intelligence, this paper emphasizes the necessity of validating its models across a spectrum of populations to prevent bias and achieve equitable healthcare outcomes. Finally, the importance of international collaboration is illustrated, showcasing its role in sharing insights and strategies to overcome global access barriers in medical imaging. Overall, this paper offers a comprehensive overview of the challenges related to disparities in medical imaging access and proposes actionable strategies to address these challenges, aiming for equitable healthcare delivery.

## 1. Introduction

Securing access to medical imaging is crucial in the promotion and preservation of health, the prevention and management of diseases, the reduction of avoidable disabilities and premature mortality, and the realization of health equity. Regrettably, substantial discrepancies in accessing healthcare and medical imaging services persist, with a disproportionate impact on vulnerable populations.

The focus of this review is to reflect on the extent of healthcare inequities and evaluate the impact of the strategies employed to address global disparities in breast cancer care. Primarily, this paper appraises the impact of scientific mobility in raising awareness and the profile of underserved populations through direct clinical contact and representative inclusion in data capture that shapes future healthcare delivery. The subsequent sections further explore the significance of cultural competency achieved through scientific mobility, emphasizing adaptation of communication techniques in alignment with cultural context, and finally translation to policy change with appropriate deployment of teleradiology and remote support services. The major contribution of this work is to provide scalable examples of solutions for early global cancer detection and timely evidence-based management in line with local needs.

Cancer is potentially the most manageable disease, and it is currently the first or second most common contributor to premature mortality in most countries of the world. Furthermore, cancer incidence is predicted to double between 2020 and 2070 [1]. The greatest increases are predicted in lower-resource settings, and survival rates in developing countries are often half those of developed countries [2,3]. Breast cancer accounts for 15.5% of all cancer deaths globally and it is the world’s most prevalent cancer. At the end of 2020, 7.8 million people were living with the diagnosis of breast cancer, with a mean global incidence of 47.8/100,000 and mortality of 13.6/100,000 [4]. The risk factors for breast cancer are more prevalent in developed countries and the incidence of breast cancer is increasing globally, in line with global development. Mortality rates in countries with a high human development index are low compared to the developing world. Most strikingly, South Korea and Fiji have a similar incidence of breast cancer at approximately 66.0/100,000; however, the mortality rate in South Korea is 6.4/100,000, compared with 41.0/100,000 in Fiji. Nevertheless, breast cancer is one of the most preventable and curable diseases: it can be detected using imaging in the subclinical stage and judicious use of medical imaging in breast cancer screening programs has been shown to have positive effects on morbidity and mortality, offsetting the risks associated with investigations [5,6]. Therefore, the reasons behind the disparities are multifactorial, with imaging playing a key role in detection and treatment [4,7]. One major issue worldwide is the lack of access to healthcare services in low-income and marginalized communities [2,7,8]. These populations often face a variety of barriers to accessing care, including lack of transportation, limited health insurance coverage, and inadequate availability of healthcare providers in their area. As a result, they may not receive timely preventative interventions or treatment for illnesses, leading to more severe health problems [9].

Access to medical imaging, such as mammography, ultrasound, and Magnetic Resonance Imaging (MRI), is also an area where inequalities exist [7]. Some patients may face long wait times for imaging services or are unable to access the services at all due to geographic or financial constraints. Such delays in diagnosis and treatment can significantly impact health outcomes for breast cancer patients.

Furthermore, there are also racial and ethnic disparities in access to medical imaging, with marginalized and low-income populations and ethnic minorities not receiving the information for timely preventative care or the treatment they need, leading to more severe health complications [10].

Studies have shown that African American and Hispanic patients are less likely to receive certain imaging procedures compared to their white counterparts, even when controlling for factors such as age, sex, and insurance status [11]. This may be due to implicit biases among healthcare providers or structural barriers within the healthcare system.

Thus, inequalities in access to care and medical imaging continue to persist, with low-income and marginalized communities and certain racial and ethnic groups facing the greatest barriers. Addressing these disparities will require a comprehensive approach that tackles the individual and systemic factors contributing to unequal access to care [8].

Radiologists can play a key role in reducing inequalities in access to care by bridging the gap between awareness and action to ensure individuals appreciate the significance of screening and have access to and utilize these services [7].

**Figure 1:**
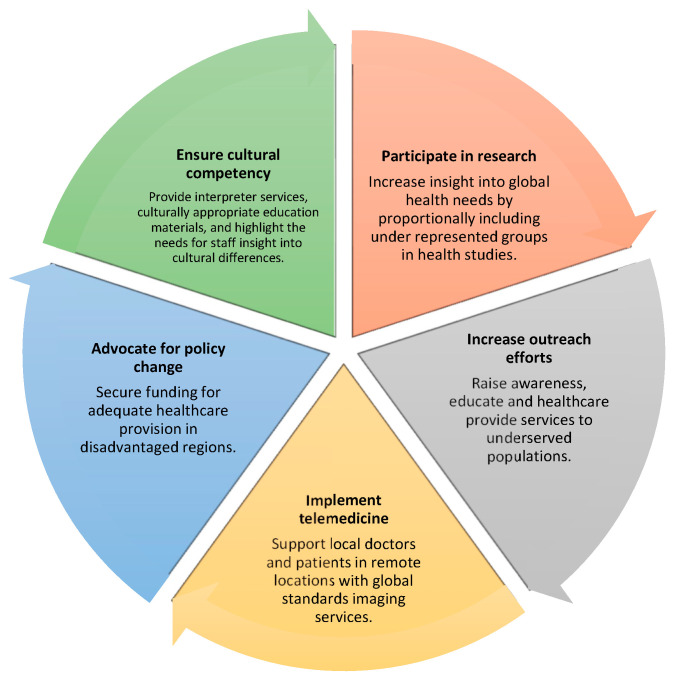
Main strategies that radiologists may implement to lessen disparities in medical imaging.

This review article’s main objective is to critically assess efforts made to lessen disparities, focusing on breast cancer screening, and to review different strategies that radiologists may implement to address this (Figure 1).

## 2. Fund Scientific Mobility and Include Under-Represented Countries in Studies and Artificial Intelligence (AI) Data Capture

In health care policies, addressing intricate challenges necessitates collaborative efforts and shared approaches to identify best practices, standardize them, and subsequently disseminate them for the enhancement of patient care [12]. Scientific mobility is defined as the physical and intellectual exchange of radiologists and researchers across international borders, encompassing participation in exchange programs, fellowships, collaborative research, and virtual learning opportunities. This initiative aims to foster a global exchange of knowledge, skills, and practices in radiology, significantly benefiting both the individuals involved and the broader medical community [13].

Over the past decade, international scientific mobility has witnessed significant growth in both the European Union (EU) and the United States, encompassing medical students, academic faculty members, and postgraduate medical trainees [13,14]. This growth aligns with the EU’s ambition to become a leading knowledge-based economy, and the US universities’ prominence in global higher education [15]. As part of this vision, the EU has issued recommendations to promote mobility, emphasizing a broader perspective encompassing enhanced job opportunities, reduced poverty levels, and the free movement of people and ideas, implementing guidelines that facilitate the mobility of researchers across various European academic centers [15]. However, the implementation of effective scientific mobility programs is fraught with challenges, primarily due to the scarcity of funding and institutional limitations [16]. Implementing policy changes necessitates robust leadership, revised governance frameworks, and heightened institutional independence. Within the radiology domain, global entities like the Radiological Society of North America (RSNA) and the European Society of Radiology (ESR) can assume a pivotal role in engaging local centers in international scientific initiatives [13]. By offering support and resources, these societies can ensure that individuals from underrepresented regions are included in global radiology initiatives. This inclusion not only enriches the individuals’ expertise but also contributes to a more comprehensive understanding of medical imaging and diagnosis worldwide [13]. Moreover, international training opportunities facilitated by these societies contribute to breaking down barriers and building bridges between different cultures and healthcare systems. The exchange of knowledge, techniques, and experiences between radiologists from various parts of the world enriches the collective understanding of medical imaging and diagnosis, leading to more comprehensive and inclusive healthcare solutions. This kind of scientific mobility could be co-funded by departments through the clinical income trainees generate by spending a percentage of their time delivering clinical radiology aligned to the host unit’s financial strategy [16].

Academic institutions offer fellowships in global radiology, focusing on training radiologists in addressing healthcare needs in low-resource settings [17]. These fellowships often include rotations in underserved areas, both domestically and internationally.

Exchange programs between institutions in high-income and low-income countries enable radiologists to experience healthcare delivery in vastly different contexts. For instance, the exchange program between the Massachusetts General Hospital and institutions in sub-Saharan Africa [18] allows participants to engage directly with the challenges of delivering radiology services in resource-limited settings: this firsthand experience is crucial in forming advocates who understand the nuances of healthcare disparities and can effectively lobby for policy changes that promote diversity and cultural competence in healthcare. The mobility of researchers and clinicians facilitates the inclusion of remote and underrepresented communities in clinical studies indeed: such inclusion is crucial to ensure that research outcomes are generalizable and applicable to diverse populations.

A program called “Radiologists Without Borders” [19] sends radiologists to underserved areas where they not only provide essential diagnostic services but also engage in teaching and capacity-building activities. By working in diverse cultural settings, these radiologists gain a deeper understanding of the unique healthcare challenges faced by these communities. The program has been instrumental in advocating for improved diagnostic resources in these regions, directly influencing policy changes aimed at increasing resource allocation. Radiologists and researchers involved in such initiatives can share their experiences and strategies through publications, conferences, and policy discussions, thereby contributing to the formulation of comprehensive and inclusive imaging guidelines.

In addition to being the first step toward policy changes for the radiology of tomorrow, mobility remains a crucial factor in the personal growth and future employability of medical researchers, promoting the appreciation of diversity and the ability to effectively engage with and embrace other cultures, which is fundamental for fostering high-quality doctor-patient relationships. Working in different cultural contexts enhances radiologists’ ability to communicate effectively with patients from various backgrounds: such improved communication is essential for advocating for policies that are sensitive to cultural differences, thereby enhancing patient care and compliance with screening programs. Moreover, scientific mobility fosters international collaborations, leading to the exchange of best practices and innovative approaches to cancer imaging: such exchanges can inspire new policy initiatives as learning from countries where successful breast cancer screening programs have been implemented can inform policy changes in regions struggling with accessibility and affordability issues [20].

## 3. Ensure Cultural Competency

Cultural competence is a key aspect of providing high-quality healthcare to diverse patient populations. The concept of cultural competence involves understanding the unique cultural, social, and linguistic needs of patients and tailoring healthcare services accordingly [21]. There is growing recognition of the importance of cultural competence in healthcare, including in radiology [22].

There are several strategies that radiologists can use to enhance their cultural competence and provide culturally sensitive care to diverse patient populations. One of the most important strategies is ongoing cultural education and training: this involves learning about language, customs, traditions, and beliefs of the patient population they serve [23]. By doing so, radiologists can better understand the cultural context of their patients and tailor their services to meet their unique needs. Cultural education and training can take many forms, including attending cultural competency workshops, participating in online courses, and reading relevant literature.

One way that radiologists can work towards cultural competence is through cultural sensitivity training [24,25]. Such training may include learning about cultural beliefs and values, communication styles, and health practices that are specific to different cultures. Recently, Davis KM. et al. [25] published the educational strategies to achieve equitable breast imaging care in diverse populations such as the US, where significant health disparities persist in breast imaging. Validated strategies include diversifying the breast imaging workforce, understanding the needs of distinct groups in a diverse population, cultural sensitivity and bias training, and fostering awareness of the existing issues in screening mammography access, follow-up imaging, and clinical care. Currently, there is no centralized radiology curriculum focusing on breast health disparities available to residents, breast imaging fellows, or practicing breast radiologists but, by understanding these differences, radiologists can better tailor their care to meet the needs of patients from diverse backgrounds.

Developing culturally sensitive communication also means that radiologists should be aware of their own cultural biases and be able to communicate effectively with patients from diverse backgrounds. This can include using appropriate language and interpretation services, as well as being aware of nonverbal communication and cultural differences in communication styles.

Several organizations have developed resources and guidelines to help radiologists improve cultural competence. The American College of Radiology (ACR) has established a Diversity and Inclusion Commission, which provides guidance on strategies to increase diversity and promote cultural competence in radiology practices. The RSNA has also developed resources and guidelines to help radiologists provide culturally competent care.

Radiologists can benefit from cultural sensitivity training to address health disparities experienced by minority populations. Developing an understanding of the cultural differences and similarities among minority patients is vital for establishing a trustworthy patient-provider relationship. To promote behavioral changes institutionally, imaging centers can implement cultural sensitivity training for physicians, trainees, nurses, technologists, and front-desk staff [26]. For gender and sexual minority patients, breast radiologists should use appropriate transgender terminology and breast imaging centers can provide gender-neutral facilities to create an inclusive and respectful environment [26].

Clinicians also require cultural sensitivity and knowledge of screening mammography regimens unique to the LGBTQ+ community to effectively address health disparities in these populations [24]. Recently, Corso et al. [27] conducted a systematic review to evaluate the impact of breast cancer on transgender populations, revealing that male-to-female individuals exhibited a higher risk of developing breast cancer compared to cisgender men. Although the risk was lower than that observed in cisgender women, it remained substantial. Given the absence of established guidelines for breast cancer prevention specific to transgender populations, individuals undergoing gender transition may benefit from regular breast or chest examinations. This recommendation underlines the importance of culturally sensitive healthcare practices that consider the unique needs and vulnerabilities of transgender individuals within the LGBTQ+ community. As clinicians work to bridge health disparities in this population, understanding these risk factors and advocating for tailored screening approaches becomes paramount.

To provide equitable care to diverse patient populations, healthcare providers must understand both conscious and unconscious biases. Conscious biases are intentional, while unconscious biases occur when individuals unknowingly rely on assumptions or stereotypes to make decisions about individuals, groups, or situations [10]. Unconscious biases are pervasive and can worsen health disparities and result in substandard breast care despite positive intentions from providers [10]. The Implicit Association Test is an effective tool that can be accessed online to test one’s own unconscious bias [8]. Additionally, providers should implement evidence-based medical practices and avoid stereotypes, and institutions can offer formal education to reduce the effects of unconscious bias [10].

Another strategy for enhancing cultural competence is diversifying the radiology workforce [28]. To promote a more diverse workplace that reflects the patient population, it is critical that radiology environments are both diverse and free from hostility, exploitation, and unequal treatment. Strategies to increase diversity in the breast imaging workforce include recruiting and retaining a more diverse population of radiology residency and breast imaging fellowship applicants, staff in breast imaging facilities, and promoting an inclusive workplace [13,22].

Finally, modification of the imaging suite is an important strategy to enhance cultural competence. For instance, including equipment and facilities that accommodate patients of different body types or religious beliefs, such as the use of female-only imaging rooms for certain patients [23]. Provision of culturally appropriate environments can help patients feel more comfortable and reduce anxiety during imaging procedures.

## 4. Improve Communication

Radiologists play a critical role in ensuring that patients receive appropriate medical imaging and that the results of these tests are communicated clearly to patients and other members of the healthcare team, helping to decrease discrepancies in access to medical imaging by ensuring that interpretation services are available when they are needed.

This is particularly important in communities where patients may not speak the national language as their first language or where there may be cultural or linguistic barriers to accessing healthcare services. Moreover, as many patients may feel overwhelmed or confused by the technical language used in radiology reports, radiologists can help to demystify this language by providing easy-to-understand patient education materials that are translated into languages commonly spoken in their communities. Communication barriers, including language gaps and inadequate patient understanding of their results, can obstruct access to care and follow-up [10]: for instance, US patients with limited English proficiency have been found to receive less health care than English-speaking patients [29]. In breast cancer care, communication gaps negatively impact follow-up of abnormal breast imaging findings [30].

Nowadays, hospitals and healthcare institutions are required to provide free, qualified language services, such as telephone or video interpreting services [8]. The use of trained interpreters can lead to increased use of health services, including screening rates, reduced inappropriate medical tests, increased compliance, and patient satisfaction [31].

Moreover, there are positive results showing the importance of collaborating with community organizations to provide educational content that is culturally and linguistically appropriate for minority groups (African American, African-born, Chinese, Latina, and Muslim women, as well as LGBTQ individuals) facing barriers to uptake of screening mammography [4].

In the context of modern healthcare, there have been notable advancements in addressing language barriers and improving communication with diverse patient populations. The emergence of mobile translation technologies presents a promising avenue to partially alleviate the challenges associated with the lack of interpreters: these innovative tools leverage the power of artificial intelligence and natural language processing to facilitate real-time translation between healthcare providers and patients who speak different languages [31]. Mobile translators offer a practical solution for bridging communication gaps, especially in situations where on-site interpreters might be unavailable or logistically challenging to arrange. These digital platforms can provide immediate language assistance, allowing medical professionals to interact with patients effectively and ensure that crucial information is accurately conveyed. While they may not fully replace the nuanced cultural understanding and context that human interpreters provide, mobile translators represent a valuable supplement to interpretation services, particularly in scenarios where timely communication is critical.

Nevertheless, it is important to acknowledge that the integration of mobile translators into healthcare settings should be approached with careful consideration [32]. Factors such as patient privacy, data security, and the potential for misunderstandings due to linguistic intricacies need to be thoroughly evaluated. Furthermore, the utilization of mobile translators should not undermine the broader goal of fostering a more inclusive and patient-centered healthcare environment, where human connections and cultural sensitivities are also promoted [31].

In conjunction with the availability of trained interpreters and the use of mobile translators, there is a growing emphasis on improving written and verbal communication processes with patients from diverse backgrounds. Efforts to ensure that patient letters, forms, and educational materials are easy to comprehend are instrumental in facilitating clear communication and fostering patient engagement. This is particularly pertinent in scenarios involving informed consent forms for clinical trials: the comprehensive understanding of these forms is crucial to encourage the participation of individuals from minority communities in clinical research. Failure to address communication difficulties could inadvertently lead to a skewed representation of the population in clinical trials, resulting in scientific findings and guidelines that lack the necessary diversity and relevance.

Making patient education resources available before and after clinical appointments can also improve patient understanding. In 2020, the American College of Radiology published the “Talking to Patients About Breast Cancer” toolkit, which provides CME credits to physicians and educational materials to both physicians and patients, including resources in Spanish [33]. Mammography and sonography technologists, who spend the most time with patients during visits, can be trained to improve communication and patient comfort. Ensuring a gender-neutral and diverse workplace also helps in conveying the breast center’s values to patients and employees.

Radiologists have been historically characterized as “doctor-to-doctor” consultants who are distanced from patients [34]. Young radiologists must expand their role from image interpretation to active participation in direct patient care. This requires embracing increased patient-facing interactions and producing reports aimed at patients. Despite evolving technology, personalized care is challenging to maintain without meaningful doctor-patient connections. However, limited time and medical staff make patient-centered care difficult. To address this, patient navigators can facilitate communication and understanding, bridging the gap between patients and the healthcare system [35]. AI also aids by expediting image analysis, potentially allowing radiologists to focus more on patient interactions [36]. Embracing these changes allows radiologists to reestablish a patient-centered approach to healthcare.

The RSNA reported a shift away from direct patient-provider engagement projects [13] that has diminished the perception of the central role radiology plays in patient care, as shown in a survey among 694 RSNA members [37]: 73% indicated that time constraints or heavy workloads often hindered direct communication with patients, with only 31% stating that their practices consistently emphasized the significance of radiology in the broader context of patients’ healthcare. This deficiency in communication exacerbates healthcare disparities, as demonstrated by an ESR analysis [38], which highlighted a strong link between direct interaction between radiologists and patients and enhanced clinical effectiveness.

Innovative application of AI for breast cancer diagnosis challenges the way doctors communicate radiological findings. A recent systematic review [39] explored patients’ psycho-cognitive perspective on AI and the doctor-patient interaction in cancer diagnosis. Despite analyzing 517 papers, most articles highlighted AI’s image interpretation success but downplayed patient trust and patient-centered communication, an overlooked concern in the medical literature. While AI aids doctors in making a diagnosis, its use does not consistently translate to effective patient communication and trust.

In conclusion, it is important to underscore that radiology’s scope extends beyond image interpretation to encompass the holistic care of patients. This recognition acknowledges that radiological education transcends the imparting of formal technical knowledge alone, as radiologists are entrusted with the vital tasks of effectively communicating diagnoses and considering patients’ values and preferences within the diagnostic process. This is becoming increasingly pertinent with the widespread adoption of healthcare apps, which grant patients direct access to their radiology reports.

## 5. Advocate for Policy Changes

Another approach to decrease disparities in access to cancer imaging is to develop policies and procedures that are sensitive to cultural differences. This may include supporting scientific mobility and more routinely representing remote communities in clinical studies. Jointly, these approaches may enhance communication between radiologists and patients [36].

For breast cancer, governments and healthcare systems across the globe have taken proactive measures to ensure equal access to screening services, aiming to remove barriers and enhance healthcare infrastructure for underserved populations [40,41,42]. Legislative actions have played a pivotal role in promoting breast cancer screening and narrowing gaps in accessibility. For instance, in some regions, legislation has mandated insurance coverage for essential screening modalities like mammography, thereby increasing affordability and accessibility for all individuals [43,44].

Although numerous national radiology organizations have published position statements on the topic of diversity, action has been slow [10]. A joint co-sponsored national conference on disparities in radiology could promote collaboration among radiologists from various subspecialties to develop potential solutions. Many national organizations, including radiological societies, generate image-based screening recommendations, but several of these recommendations neglect to consider diversities of populations [10].

In the era of AI, it was recently pointed out that there is a considerable racial bias in algorithms commonly used in the health systems, and that there is a predilection towards the prediction of health care expenses rather than treating sickness [45]. There is growing concern that algorithms could perpetuate gender and racial inequality through the people who create them or through the training data [45,46,47,48]. Indeed, models were often trained on relatively homogeneous datasets, thus, populations that are underrepresented have limitations on generalizability and pose a danger of biased AI-based conclusions. One example is the two recent pivotal studies [49,50] from Sweden on AI in breast cancer screening that validated the efficacy of AI as a reliable adjunct or even partial substitute for human radiologists. The potential to maintain diagnostic accuracy while alleviating the radiologist’s workload is noteworthy, allowing for a higher volume of screenings within existing resource constraints, potentially reducing waiting times and increasing the accessibility of mammography to broader populations. However, both studies were based on a very specific and homogeneous population, and few similar studies on AI-enhanced mammography in different populations are currently available [51].

A previous review of the AI literature [52] found that nearly all of the top 10 databases and author nationalities were from high income countries, and US and Chinese datasets and authors were disproportionately overrepresented in clinical AI. Populations in data-rich regions, in which models are developed, benefit substantially more than data-poor regions, entrenching existing healthcare disparities.

Vigilance in the validation and calibration of AI models becomes crucial as we strive to address health inequities. Prior to the clinical deployment of AI systems, it is essential to rigorously validate their effectiveness across diverse populations. This includes conducting external validation studies that encompass a range of demographic groups, thereby accounting for variations in genetic, environmental, and socio-economic factors that can influence health outcomes. Through this process, we can identify potential biases and shortcomings in the AI models and take steps to recalibrate them accordingly [53]. Recognizing and addressing these disparities is crucial in formulating comprehensive imaging guidelines that cater to the needs of all populations. Learning from the strategies employed by radiology groups that have successfully navigated these obstacles can provide valuable insights for the development of more inclusive guidelines. National and international conferences can play an important role in promoting the sharing of state and national insurance-related barriers. For instance, most practicing radiologists probably have limited understanding of insurance coverage limitations and this lack of knowledge limits radiologists’ chances of advocating and lobbying for change [10]: an assistance in educating radiologists on disparities related to specific insurance coverage issues could help in empowering radiologists to instigate change [10].

## 6. Implement Teleradiology Services, Remote Support, and Global Involvement of Local Doctors

Teleradiology is a rapidly growing field that uses telecommunication technology to transmit radiologic images and other medical data from one location to another for the purpose of interpretation and diagnosis. Access to imaging services is a crucial component of modern healthcare, but many patients in remote or underserved areas face barriers to obtaining these services indeed. Teleradiology has recently emerged as a promising solution to this problem. Although the concept of telemedicine existed for decades, developments in technology galvanized the ability to provide this service on a large-scale basis and the COVID-19 pandemic accelerated the process [54].

Telemedicine can undoubtedly complement these efforts by enabling remote consultations, facilitating second opinions, and extending specialized knowledge to remote areas. This dual approach of leveraging teleradiology to provide access to specialized expertise while simultaneously empowering local professionals can lead to more sustainable and effective healthcare delivery in underserved regions.

One of the key benefits is the ability to provide timely interpretations for a number of radiological examinations—like mammography for breast cancer screening—even in areas where radiologists are not available on-site [51,55]. This can improve patient outcomes by enabling healthcare providers to make faster and more accurate diagnoses.

On the other hand, teleradiology cannot fully replace the importance of investments in on-site personnel. The physical presence of trained medical professionals at healthcare facilities remains crucial for comprehensive patient care, especially in cases requiring immediate attention such as contrast medium complications, complex diagnostics requiring on-table imaging review, or hands-on diagnostics and interventions. Moreover, not all diagnostic imaging modalities can readily employ the advantages of teleradiology. For instance, US examinations are inherently operator-dependent procedures, where the skill and expertise of the operator significantly influence the quality and accuracy of the results obtained. Additionally, radiographers working remotely also require tele back up from experts in their field, as well as engineering support to maintain the equipment.

Despite its benefits, teleradiology also presents potential challenges that must be addressed to ensure its effectiveness and safety. One of the main concerns is the need to ensure the security and privacy of patient data. Teleradiology involves the transmission of sensitive medical information over long distances, which can increase the risk of data breaches or other security breaches. The legal obligation to protect patients’ privacy of health data is one of the crucial priorities. Both the General Data Protection Regulation (GDPR) in EU and the Health Insurance Portability and Accountability Act (HIPAA) in US define standards and safeguards that protect patients’ health records as well as personal health information that apply to all healthcare providers, insurers, and other healthcare entities [56]. The remote healthcare provider also needs to be locally registered and legally accountable under local jurisdiction.

Another potential challenge is the need for quality control and standardization in teleradiology. The interpretation of radiologic images can be subjective, and it is important to ensure that radiologists are using standardized protocols and guidelines to ensure interpretations are of consistently high quality and meaningfully communicated [55].

Concerning breast cancer screening, the American Society of Clinical Oncology recently suggested mobile mammography as a potential tool to improve access and permit screening in low- and middle-income countries [43]. While fixed mammography units drew users primarily from financially stable backgrounds, often possessing insurance coverage, and displaying high screening adherence rates [57], mobile unit users were predominantly from underserved communities marked by transient lifestyles and living arrangements, resulting in lower adherence levels [43]. Vang et al. [58] emphasized certain deficiencies and obstacles associated with mobile units, including issues of image quality, incomplete scans, elevated recall rates, and limited integration of advanced technologies like film-screen, full-field digital, and tomosynthesis. Despite the increasing global implementation of mobile mammographic units, particularly in regions such as sub-Saharan Africa and South America, challenges tied to sociodemographic factors and recall rates persist, thereby impacting screening quality and adherence [59,60].

Finally, even teleradiology is widening the existing gap in access to care in certain circumstances. One recent study found that patients over 65 years old have the lowest odds of using telemedicine services, and Black and Hispanic patients have lower odds of using these services than their White or Asian counterparts [61]. We need to understand and resolve these discrepancies.

## 7. Conclusions

Addressing disparities in access to clinical imaging, particularly in breast cancer screening, necessitates a collaborative effort.

This review has highlighted key strategies for radiologists to help mitigate these disparities, yet there are limitations in the current research that must be acknowledged, and there are areas where future efforts could be directed.

Firstly, the implementation of cultural competence, though critical, faces challenges due to the dynamic and diverse nature of cultural identities. Continuous adaptation and updating of training programs are necessary to keep pace with evolving societal norms. Future research should focus on developing and validating culturally sensitive training modules and evaluating their impact on patient outcomes.

Additionally, while promoting scientific mobility among radiologists is beneficial for creating an inclusive workplace, it is important to consider the practical barriers such as varying licensure requirements and professional recognition across regions. Future initiatives could explore streamlined processes for international collaboration and exchange.

The use of mobile translation technologies and AI in addressing language barriers shows promise, but the effectiveness and accuracy of these technologies in conveying nuanced medical information remain a concern. Future research should investigate the reliability of these tools in clinical settings and develop guidelines for their use.

Teleradiology, although a promising solution for underserved areas, raises questions about the quality of care, particularly in complex cases requiring multi-disciplinary consultation. Further research is needed to establish protocols for quality assurance and to study the long-term impact of teleradiology on patient outcomes.

By implementing these strategies and addressing the various challenges that arise, radiologists can work toward a more equitable healthcare system that serves all patients, regardless of their background or location. While the outlined strategies offer a framework for reducing disparities in clinical imaging access, future efforts should aim to refine research and innovation to address the evolving challenges in this field, assess their effectiveness, and ensure they are adaptable to diverse healthcare environments.

## Abbreviations

ACR	American College of Radiology
AI	Artificial Intelligence
CME	Continuing Medical Education
ESR	European Society of Radiology
EU	European Union
GDPR	General Data Protection Regulation
HIPAA	Health Insurance Portability and Accountability Act
LGBTQ+	Lesbian, Gay, Bi, Trans, Queer and Others
MRI	Magnetic Resonance Imaging
RSNA	Radiological Society of North America
